# The Effect of *Curcuma longa* on Inflammatory Mediators and Immunological, Oxidant, and Antioxidant Biomarkers in Asthmatic Rats

**DOI:** 10.1155/2021/4234326

**Published:** 2021-11-12

**Authors:** Mohammad Hossein Boskabady, Fatemeh Amin, Farzaneh Shakeri

**Affiliations:** ^1^Neurogeneic Inflammation Research Center, Mashhad University of Medical Sciences, Mashhad, Iran; ^2^Department of Physiology, School of Medicine, Mashhad University of Medical Sciences, Mashhad, Iran; ^3^Non-Communicable Diseases Research Center, Rafsanjan University of Medical Sciences, Rafsanjan, Iran; ^4^Department of Physiology and Pharmacology, School of Medicine, Rafsanjan University of Medical Sciences, Rafsanjan, Iran; ^5^Natural Products and Medicinal Plants Research Center, North Khorasan University of Medical Sciences, Bojnurd, Iran; ^6^Department of Physiology and Pharmacology, School of Medicine, North Khorasan University of Medical Sciences, Bojnurd, Iran

## Abstract

The effects of *Curcuma longa* (*C. longa*) on total and differential WBC, inflammatory and immunologic mediators, and oxidant and antioxidant biomarkers in bronchoalveolar lavage fluid (BALF) of rats model of asthma were assessed. Animals were divided to 5 groups including control (C), asthma (sensitized to ovalbumin), and asthmatic groups treated with 0.75, 1.50, and 3.00 mg/ml *C. longa* (CL) and 1.25 *μ*g/ml dexamethasone (D) (8 rats in each group). Total and differential WBC count, concentrations of phospholipase A_2_ (PLA_2_), total protein (TP), interferon-gamma (IFN-*γ*), interleukin-4 (IL-4), immunoglobulin E (IgE), NO_2_, NO_3_, malondialdehyde (MDA), superoxide dismutase (SOD), catalase (CAT), and thiol in BALF were assessed. Total and most differential WBC counts and BALF levels of PLA_2_, TP, IgE, IL-4, and oxidants in asthma group were higher but antioxidants and IFN-*γ* levels as well as IFN-*γ*/IL-4 ratio were lower than control group (*p* < 0.001 for all cases). Total WBC and levels of PLA_2_, IgE, NO_2_, and NO_3_ were significantly reduced following treatment with *C. longa*, compared to asthma group (*p* < 0.001 for all cases). In groups treated with dexamethasone and two higher concentrations of *C. longa*, neutrophil and eosinophil counts as well as TP, IL-4, and MDA levels were significantly decreased but IFN-*γ*, IFN-*γ*/IL-4 ratio, and antioxidants were increased (except IFN-*γ*/IL-4 ratio), compared to asthma group (*p* < 0.05 to *p* < 0.001). Compared to dexamethasone, *C. longa* exerted more pronounced effects on lung inflammation, oxidative stress, and immune system in asthmatic rats.

## 1. Introduction

*Curcuma longa* L. *(C. longa)* is a perennial herb of the Zingiberaceae family (ginger), with the common name Turmeric. The plant is used as a traditional cure in India, China, and other Southeast Asian nations for the treatment of asthma and common cold [1]. In Ayurvedic medicine, turmeric is a well-known remedy for several respiratory problems such as asthma and allergy, runny nose, cough, and sinusitis [2]. Moreover, it is used not only as a spice in food preparation as the major ingredients of curry and mustard but also as a functional food due to its potential health benefits [3].

*C. longa* contains various constituents including terpenes, phenolic compounds, steroids, fatty acids, and other compounds. The level of curcumin, the main curcuminoid of *C. longa*, varies between varieties and cultivation locations and conditions (1.28 to 6.6%) and North Indian plains (0.61 to 1.45%), on a dry weight basis [4].

*C. longa* contains carbohydrates, moisture, protein, fat, and minerals. The essential oil of rhizomes the plant using steam distillation contains sesquiterpenes, zingiberene, a-phellandrene, cineol, sabinene, and borneol. It includes curcumin I (94%), curcumin II (6%), and curcumin III (0.3%), responsible for the vibrant yellow color of turmeric [5].

Various anti-inflammatory [6], antiasthmatic [7], relaxant [8, 9], and antioxidant [10] effects were described for this plant. The effect of turmeric on ovalbumin (OVA)-sensitized mice was demonstrated to be antioxidant [11] and had immunomodulatory effects by altering the balance of CD4‏^+^ CD25^+^ regulatory T cells (Tregs)/T-helper (Th), [12]. Treatment with methanolic extract of *C. longa* (200 mg/kg, for 14 days) stimulated innate and adaptive immunity in mice. The effect of the extract on adaptive immunity was evaluated by immunizing and challenging the mice with sheep red blood cells (sRBCs) on days 7 and 14, respectively. *C. longa* enhanced the adaptive immunity by increasing leukocyte number, antibody titer, spleen index, and delayed-type hypersensitivity response [13]. In another study, *C. longa* root aqueous extract standardized was indicated to stimulate NO production in RAW264.7 macrophages [14].

Asthma is a chronic inflammatory airway disorder involving inflammatory cells such as eosinophils, mast cells, T-lymphocytes, epithelial cells macrophages, and neutrophils [15]. These inflammatory cells produce inflammatory mediators, which play a key role in pulmonary inflammation and asthma [16]. The first line of defense against inhaled environmental oxidants, and endogenous oxidants, is the high concentrations of antioxidants in the epithelial lining fluid in the lung [17]. Alterations in the antioxidant/oxidant balance in bronchoalveolar lavage fluid (BALF) in asthma can indicate a continuous inflammation procedure. There are reports regarding the imbalance in Th1/Th2 ratio toward increased Th2 activity, in asthma [18]. Th2 cells secrete IL-4, IL-5, and IL-13, which lead to allergic inflammatory diseases. Th1 produces IL-2 and IFN-*γ* which in turn inhibit Th2 activities [19]. Therefore, increasing IFN-*γ*/IL-4 or Th1/Th2 ratios could be considered a treatment approach for asthma.

Although there are several studies on the immunological and antioxidant effects of *C. longa*, the effect of the plant on immunological changes and oxidative stress in asthma was not fully addressed yet. Therefore, in the present study, the potential anti-inflammatory, antioxidant, and immunomodulatory effects of the extract of *C. longa* hydroethanolic on sensitized rats were evaluated by measuring total and differential WBC count, PLA_2_, TP, IgE, IFN-*γ*, IL-4, and oxidant and antioxidant biomarker levels in BALF.

## 2. Materials and Methods

### 2.1. Asthma Induction in Rats

Male Wistar rats (age 8-9 weeks and weight 200–250 g) model of asthma was induced by three intraperitoneal (i.p.) injections of 1 mg/kg chicken egg albumin (Ovalbumin = OVA) in 0.9% sterile saline + 100 mg Al(OH)_3_, on days 1, 2, and 3. Animals were then exposed to aerosolize 1% OVA for 20 min/day, on days 6, 9, 12, 15, 18, and 21 as previously described [20].

Animals were obtained from Animal House, School of Medicine, Mashhad University of Medical Sciences, Mashhad (MUMS), Iran, and kept in a temperature-controlled room (22 ± 2C°) with 12 h light/12 h dark cycles and had free access to food and tap water. Ethics Committee of MUMS approved the study (ethics allowance no. 921249), and the regulations of the Institute of Laboratory Animals Resources Commission on Life Sciences were followed in animal handling.

### 2.2. Plant Materials and Extraction

**Figure 1:**
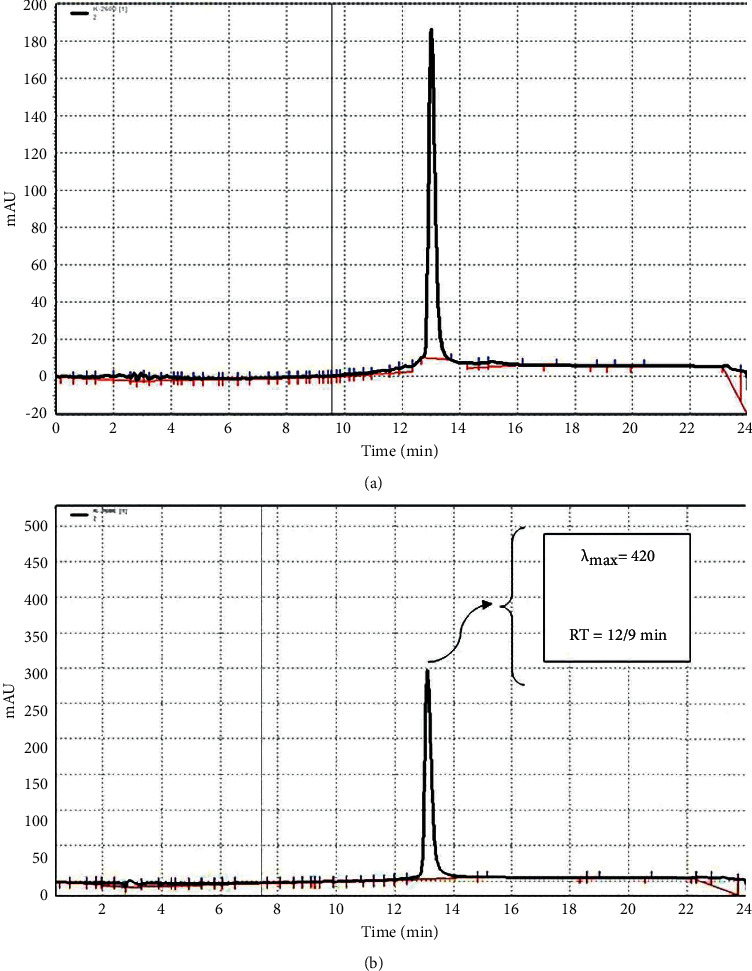
RP-HPLC of (a) the extract of *C. longa* and (b) curcumin (8).

*C. longa* rhizomes (100 grams) were purchased from a local herbal market in Mashhad, Khorasan Razavi province, Iran. Then, it was ground to powder and extracted with ethanol (96%). The resultant extract was concentrated under low pressure in an Eyela (Heidolph, Schwabach, Germany) rotary evaporator. The extraction yield was 14% [21]. The qualitative and quantitative characteristics of curcumin in the extract [8] was determined as previously described (Figure 1).

### 2.3. Experimental Animal Groups

Animals were randomly divided into the following groups (*n* = 8 in each group):

- Control or saline-treated rats (group C)
- Asthmatic rats (OVA sensitized, group A)
- Asthmatic animals treated with different concentrations (0.75, 1.5, and 3 mg/ml) of *C. longa* (groups CL 0.75, CL 1.50, and CL 3.00, respectively)
- Asthmatic animals treated with dexamethasone (1.25 *μ*g/ml) (group A + D)

In treated animals, the extract of *C. longa* and dexamethasone were added to the drinking water during sensitization period. On average, every rat consumed 40 ml of drinking water each day, and no significant difference was made between different groups in water intake.

### 2.4. Evaluation of WBC Count (Total and Differential), Inflammatory Mediators, and Cytokines Levels in the BALF

At the end of the sensitization period, rats were deeply anesthetized by i.p. injection of 50 mg/kg ketamine. The animals were sacrificed by a competent researcher with minimum pain, suffering, and distress. The method was performed according to Annex IV of the guidelines from Directive EU/2010/63 of the European Parliament guideline. The trachea and lungs were then removed after the chest was opened. The right lung was lavaged five times with 1 ml saline (a total of 5 ml).

Total WBC was counted in 1 ml BALF after staining with Turk solution in duplicate using a hemocytometer as previously described [22]. Under light microscopy, differential WBC was determined using morphological criteria by counting 100 cells and calculating the percentage of each cell type in a smear of the cells stained with Wright-Giemsa [23].

The remaining BALF was centrifuged at 2500×*g* at 4°C for 10 min, supernatant was removed, and inflammatory mediator (PLA_2_), oxidant and antioxidant biomarkers, cytokine, and immunological (IFN-*γ*, IL-4, and IgE) levels were measured according to the method described previously [20].

### 2.5. Evaluation of Oxidative Stress Biomarkers in BALF

The products of NO metabolism (NO_2−_/NO_3−_) [24], malondialdehyde (MDA) levels [25], superoxide-dismutase (SOD) [21], and catalase (CAT) [23] activities, and total thiol concentration [20] in the BALF supernatant were measured using previously described methods.

### 2.6. Statistical Analysis

One-way analysis of variance (ANOVA) with Tukey-Kramer post-hoc test was used for comparisons among and within groups using InStat (GraphPad Software, USA). The data are shown as mean ± SEM. *p* < 0.05 was selected as statistical significance criterion.

## 3. Results and Discussion

### 3.1. The Changes in the Asthmatic Group

**Figure 2:**
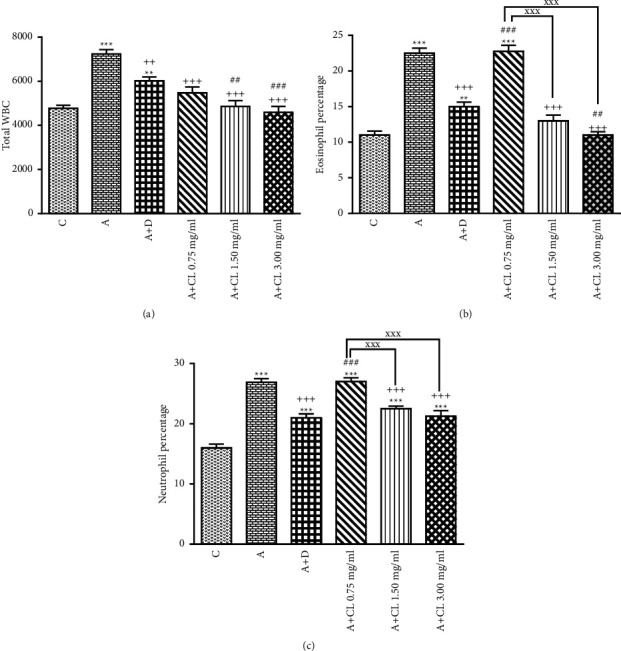
The effect of *C. longa* on total WBC number (count/ml of BALF) (a) and percentage of eosinophil (b) and neutrophil (c) in control animals (C), asthma group (A), and asthmatic groups treated with dexamethasone (D) and *C. longa* (CL) (0.75 mg/ml, 1.50 mg/ml, and 3.00 mg/ml) (for each group, *n* = 8). Data are presented as mean ± SEM. ^*∗∗*^*p* < 0.01, ^*∗∗∗*^*p* < 0.001, compared to group C. ^++^*p* < 0.01, ^+++^*p* < 0.001, compared to group A. ^##^*p* < 0.01, ^###^*p* < 0.001, compared to group D. ^xxx^*p* < 0.001, comparison among three concentrations of *C. longa*. Statistical analyses were performed using one-way analysis of variance (ANOVA) with Tukey-Kramer's post-test.

**Figure 3:**
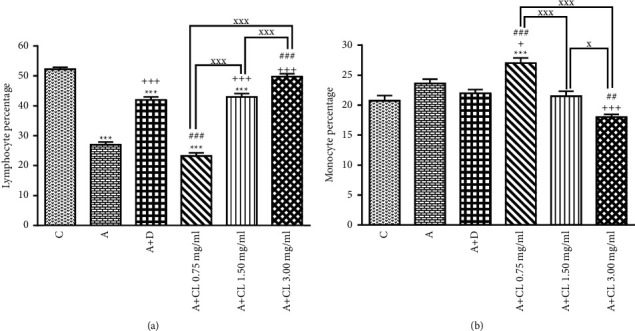
The effect of *C. longa* on percentage of lymphocyte (a) and monocyte (b) in control animals (C), asthma group (A), and asthmatic groups treated with dexamethasone (D) and *C. longa* (CL) (0.75 mg/ml, 1.50 mg/ml, and 3.00 mg/ml) (for each group, *n* = 8). Data are presented as mean ± SEM. ^*∗*^*p* < 0.05, ^*∗∗∗*^*p* < 0.001, compared to group C. ^+^*p* < 0.05, ^+++^*p* < 0.001, compared to group A. ^##^*p* < 0.01, ^###^*p* < 0.001, compared to group D. ^x^*p* < 0.05, ^xxx^*p* < 0.001, comparison among three concentrations of *C. longa*. Statistical analyses were performed using one-way analysis of variance (ANOVA) with Tukey-Kramer's post-test.

**Figure 4:**
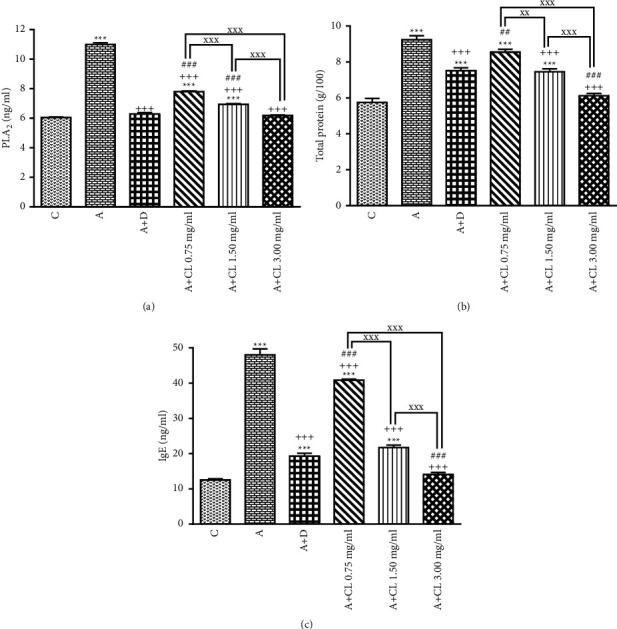
The effect of *C. longa* on TP (a), PLA_2_ (b), and IgE (c) in control animals (C), asthma group (A), and asthmatic groups treated with dexamethasone (D) and *C. longa* (CL) (0.75 mg/ml, 1.50 mg/ml, and 3.00 mg/ml) (for each group, *n* = 8). Data are presented as mean ± SEM. ^*∗*^*p* < 0.05, ^*∗∗*^*p* < 0.01, ^*∗∗∗*^*p* < 0.001, compared to group C. ^++^*p* < 0.01, ^+++^*p* < 0.001, compared to group A. ^##^*p* < 0.01, ^###^*p* < 0.001, compared to group D. ^xx^*p* < 0.01, ^xxx^*p* < 0.001, comparison among three concentrations of *C. longa*. Statistical analyses were performed using one-way analysis of variance (ANOVA) with Tukey-Kramer's post-test.

**Figure 5:**
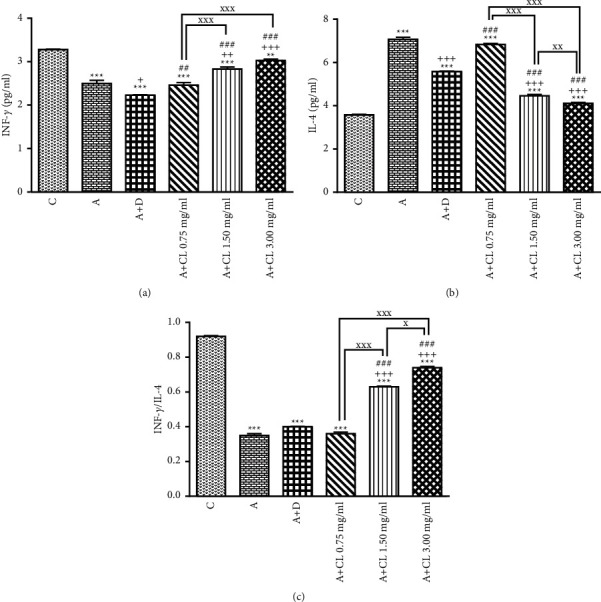
The effect of *C. longa* on IFN-*γ* (a), IL-4 (b), and IFN-*γ*/IL-4 ratio (c) in control animals (C), asthma group (A), and asthmatic groups treated with dexamethasone (D) and *C. longa* (CL) (0.75 mg/ml, 1.50 mg/ml, and 3.00 mg/ml) (for each group, *n* = 8). Data are presented as mean ± SEM. ^*∗∗*^*p* < 0.01, ^*∗∗∗*^*p* < 0.001, compared to group C. ^+^*p* < 0.05, ^++^*p* < 0.01, ^+++^*p* < 0.001, compared to group A. ^##^*p* < 0.01, ^###^*p* < 0.001, compared to group D. ^x^*p* < 0.05, ^xx^*p* < 0.01, ^xxx^*p* < 0.001, comparison among three concentrations of *C. longa*. Statistical analyses were performed using one-way analysis of variance (ANOVA) with Tukey-Kramer's post-test.

**Figure 6:**
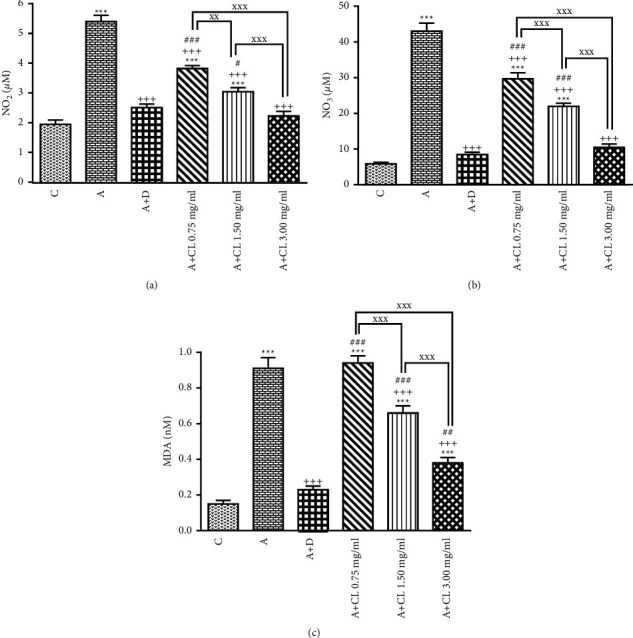
The effect of *C. longa* on NO_2_ (a), NO_3_ (b), and MDA (c) concentration in control animals (C), asthma group (A), and asthmatic groups treated with dexamethasone (D) and *C. longa* (CL) (0.75 mg/ml, 1.50 mg/ml, and 3.00 mg/ml) (for each group, *n* = 8). Data are mean ± SEM. ^*∗∗∗*^*p* < 0.001, compared to group C. ^+++^*p* < 0.001, compared to group A. ^#^*p* < 0.05, ^##^*p* < 0.01, ^###^*p* < 0.001, compared to group D. ^xx^*p* < 0.01, ^xxx^*p* < 0.001, comparison among three concentrations of *C. longa*. Statistical analyses were performed using one-way analysis of variance (ANOVA) with Tukey-Kramer's post-test.

**Figure 7:**
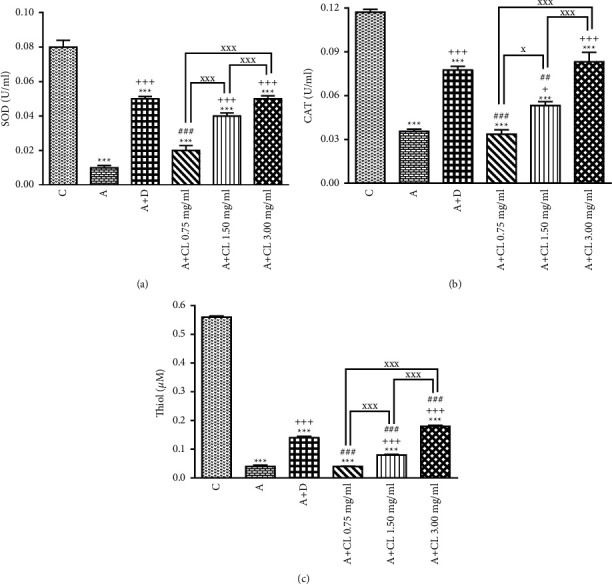
The effect of *C. longa* on SOD (a), CAT (b), and thiol (c) concentration in control animals (C), asthma group (A), and asthmatic groups treated with dexamethasone (D) and *C. longa* (CL) (0.75 mg/ml, 1.50 mg/ml, and 3.00 mg/ml) (for each group, *n* = 8). Data are mean ± SEM. ^*∗∗∗*^*p* < 0.001, compared to group C. ^+^*p* < 0.05, ^+++^*p* < 0.001, compared to group A. ^##^*p* < 0.01, ^###^*p* < 0.001, compared to group D. ^x^*p* < 0.05, ^xxx^*p* < 0.001, comparison among three concentrations of *C. longa*. Statistical analyses were performed using one-way analysis of variance (ANOVA) with Tukey-Kramer's post-test.

In asthmatic animals, total WBC count, percentages of eosinophil and neutrophil, and BALF levels of PLA_2_, TP, IgE, IL-4, NO_2_, NO_3_, and MDA were significantly higher but lymphocyte percentage, IFN-*γ* level, and IFN-*γ*/IL-4 ratio as well as SOD, CAT, and thiol levels were lower than those in nonasthmatic (i.e., group C) rats (*p* < 0.001 for all cases; Figures 2–7).

### 3.2. The Extract of C. longa Effects

Treatment of asthmatic animals with all concentrations of *C. longa* significantly reduced total WBC and BALF levels of PLA_2_, IgE, NO_2_, and NO_3_ compared to asthmatic group (*p* < 0.001 for all cases). Two higher concentrations of *C. longa* also significantly decreased neutrophil and eosinophil counts, as well as TP, IL-4, and MDA levels, but increased lymphocyte count, IFN-*γ*/IL-4 ratio, and BALF levels of IFN-*γ*, SOD, CAT, and thiol compared to untreated asthmatic group (*p* < 0.05 to *p* < 0.001; Figures 2–7).

The percentages of eosinophil and monocyte in group treated with low concentration of *C. longa*, lymphocyte percentage, and levels of PLA_2_, TP, IgE, NO_2_, and NO_3_ in groups treated with two lower concentrations of *C. longa*, as well as neutrophil percentage, IFN-*γ*/IL-4 ratio, and levels of IFN-*γ*, IL-4, MDA, SOD, CAT, and thiol in groups treated with all concentrations of *C. longa* were significantly different compared to control group (*p* < 0.01 to *p* < 0.001; Figures 2–7).

### 3.3. Dexamethasone Effects

Total WBC, percentages of eosinophil and neutrophil, and levels of PLA_2_, TP, IgE, IFN-*γ*, IL-4, NO_2_, NO_3_, and MDA were decreased but lymphocyte percentage and SOD, CAT, and thiol levels were increased in dexamethasone-treated group compared to untreated asthmatic group (*p* < 0.05 to *p* < 0.001). However, dexamethasone did not affect IFN-*γ*/IL-4 ratio and monocyte percentage compared to asthmatic group (Figures 2–7).

In asthmatic group treated with dexamethasone, however, total WBC, percentages of eosinophil, neutrophil, and lymphocyte, and levels of TP, IgE, IFN-*γ*, IL-4, SOD, CAT, and thiol were significantly different compared to control group (*p* < 0.01 to *p* < 0.001; Figures 2–7).

### 3.4. Differences among the Effects of Various Concentrations of C. longa Extract

Eosinophil, neutrophil, lymphocyte, and monocyte percentages, IFN-*γ*/IL-4 ratio, and levels of PLA_2_, TP, IgE, IL-4, IFN-*γ* NO_2_, NO_3_, MDA, SOD, CAT, and thiol were significantly higher in asthma groups treated with two higher concentrations of the extract (1.50 and 3.00 mg/ml) compared to its low concentration (*p* < 0.05 to *p* < 0.001). The effects of high extract concentration on lymphocyte and monocyte percentages and levels of PLA_2_, TP, IgE, IL-4, IFN-*γ*/IL-4, NO_2_, NO_3_, MDA, SOD, CAT, and thiol were also significantly higher than the medium concentrations (*p* < 0.05 to *p* < 0.001).

### 3.5. Comparison of the Effect of C. longa Extract with Dexamethasone on Asthma

Treatment with all concentrations of the extract on IFN-*γ* level, treatment with its two higher concentrations on total WBC, IL-4 level, and IFN-*γ*/IL-4 ratio, and treatment with its high concentration on eosinophils, lymphocytes, and monocytes percentages and levels of TP, IgE, and thiol had significantly more marked effects compared to dexamethasone (*p* < 0.01 to *p* < 0.001; Figures 2–7). The effect of all concentrations of the extract on MDA, the effect of its two lower concentrations on PLA_2_, NO_2_, NO_3_, CAT, and thiol, and the effect of its low concentration on eosinophil, neutrophil, monocyte, and lymphocyte percentages and levels of TP, IgE, IL-4, and SOD were significantly lower than that of dexamethasone (*p* < 0.05 to *p* < 0.001; Figures 2–7).

## 4. Discussion

After OVA sensitization, a rat model of asthma was produced; in asthmatic animals, total WBC, percentages of eosinophil and neutrophil, and BALF levels of PLA_2_, TP, IgE, IL-4, NO_2_, NO_3_, and MDA were increased while lymphocyte percentage, IFN-*γ*/IL-4 ratio, and IFN-*γ*, SOD, CAT, and thiol levels were decreased compared to control animals. These results demonstrated asthma induction in rats.

Previously, increased total WBC and neutrophil and eosinophil percentage in lung lavage of OVA-sensitized rats were shown [23, 26]. Increased percentages of eosinophils were also demonstrated in sensitized animals and asthmatic patients that correlated with the severity of asthma and supported the results of the present study [27]. Activated eosinophils are critical in allergic reactions and induce respiratory hyperactivity in asthma [28].

In etiology of asthma and COPD, lymphocytes play a crucial role. A greater number of CD8^+^ type-1 T-lymphocytes and lung macrophages indicate chronic inflammation of airways [29]. The present investigation found that the lymphocyte percentage of asthmatic animals in BALF has decreased. Moreover, in a previous study which used a similar sensitization protocol to that of the current study, increased absolute lymphocyte count was observed. Therefore, reduction of lymphocyte percentage could be due to increased total WBC of asthmatic animals [27]. If the absolute lymphocyte count was considered, it would be higher in the asthmatic rats compared to control group.

There has been an increase in the amount of neutrophils in lung lavage of asthma patients and a significant link between inflammation of the airways and severity of asthma [30, 31]. Neutrophils include significant cytokine sources including tumor necrosis factor-*α* (TNF-*α*), interleukin-1*β* (IL-1*β*), IL-6, and IL-8. Activated neutrophils are able to release human neutrophil elastase (HNE) and myeloperoxidase (MPO) that exacerbate and prolong asthma symptoms and contribute to the airway inflammation [32].

Alterations in monocyte numbers in the peripheral blood are associated with asthma attacks induced by allergic reactions. Activation of monocytes also contributes to asthma pathogenesis by releasing reactive nitrogen species, total free radicals, and proliferation of Th2-like lymphocytes in patients with asthma [33].

Both animal and human studies have shown increased PLA_2_ and TP in asthma [27, 34]. Consequently, higher levels of PLA_2_ and TP in the BALF of asthmatic rats also confirm the induction of an animal model of asthma in the present study. PLA_2_ has a significant function in asthma pathogens through the development of eicosanoid formation, dendritic cells maturation and migration, T cells proliferation, and production of cytokines and chemokines by monocytes, macrophages, neutrophils, and eosinophils [35].

The findings from the current investigation were comparable to those in earlier studies that induced asthma using a similar sensitization method [36, 37] which also confirmed animal sensitization with changes in IL-4, IFN-*γ*, and IFN-*γ*/IL-4 ratio. *In vivo* and *in vitro* studies have also shown increased IL-4 but reduced IFN-*γ* and IFN-*γ*/IL-4 in OVA-sensitized guinea pigs [38]. Increased release of Th2 cytokines mainly IL-4, IL-5, and IL-13 and decreased release of IFN-*γ* as a Th1 cytokine also contribute in the onset and progression of asthma [29].

In accordance with the results of this study on indicators of oxidant/antioxidants, there is clear evidence of the function of oxidative stress in inflammation of airways in asthmatic conditions [39]. Increasing production of oxidants in asthmatic groups in comparison to healthy controls was reported in both animal and human investigations [40]. Increased NO_2_ and NO_3_ levels in exhaled breath condensate of asthmatic patients were also associated with oxidative stress [41]; also an increased plasma MDA and amplified protein carbonyl levels were also identified in BALF and peripheral blood sample [42]. Alterations in SOD activity caused apoptosis and damaged bronchial epithelial cells, which make a considerable contribution to rehabilitation in the airways and hyper responsiveness of asthma [43]. Decreases in CAT activity possibly augment oxidative stress in the airways in asthma and perpetuate the inflammatory processes [44]. Thiol is an initial sign of oxidative stress in asthma and COPD [45]. Reduction of SOD levels [46], CAT activity, and other antioxidants, such as ascorbate, a-tocopherol, and glutathione, were shown in asthmatic patients [47], which supports the current study's findings.

Total WBC, neutrophil and eosinophil percentages, and PLA_2_, TP, IL-4, IgE, NO_2_, NO_3_, and MDA levels were significantly reduced but IFN-*γ*/IL-4 ratio, IFN-*γ*, lymphocyte, SOD, thiol, and CAT levels were increased in asthmatic rats treated with the extract of *C. longa* compared to untreated asthmatic group.

The preventive effects of *C. longa* on lung inflammatory cells, inflammatory mediators, and immunological and oxidant-antioxidant markers in asthmatic animals can be identified from these studies.

The effect of *C. longa* on several aspects of asthma was shown previously. Reduction of total and most differential WBC counts in BALF of sensitized rats treated with methanolic extract of *C. longa* [7] decreased PLA_2_ activity in BALF of OVA-sensitized mice by intranasal curcumin [48], and reduction of total protein in lung tissue homogenate of OVA-sensitized rats by *C. longa* extract [7] was documented in this regard. Curcumin has also considerably reduced BALF level of TP in a rat model of lipopolysaccharide (LPS)-induced experimental acute lung injury [49]. Moreover, treatment with curcumin suppressed the elevated level of IgE in BALF of OVA-challenged mice [50]. Moreover, asthma and allergic conjunctivitis have been improved by turmeric and curcumin therapy through regulation of Th2, IgE, and immune response to mast cells [51, 52].

Curcumin downregulated IL-4 and IL-5 levels but upregulated IFN-*γ* in the BALF of a murine model of asthma [50] and attenuated allergic airway inflammation by modifying the balance of CD4‏^+^ CD25^+^ regulatory T cells (Tregs)/T-helper (Th) in ovalbumin (OVA)-sensitized mice [53] dose-dependently. Another study showed that *C. longa* diminished OVA-induced food allergy by maintaining Th1/Th2 balance in mice [12], which supports the preventive impact of this plant on Th1/Th2 asthma imbalance as seen in this study.

Treatment with methanolic extract of *C. longa* normalized the elevated levels of nitrate and MDA in OVA-sensitized rats [7]. Curcumin and *C. longa* enhanced the activity of SOD [54] and increased CAT activity in the kidney of rats exposed to acetaminophen [55], and curcumin increased thiol concentration in a model of lung carcinogenesis induced by benzo(a)pyrene in mice [56] which was also shown. The current study's findings are supported by the previously described studies, which show that *C. longa* has a preventive therapeutic effect on asthma.

Prevention of lung inflammation and airway responsiveness by inhaled PLA_2_ inhibitor in asthma was shown previously [57]. It was also reported that inhibiting the effects of IgE is a novel strategy for blocking or ameliorating symptoms of asthma and allergy [58]. These two studies also support the preventive effect of the plant on PLA_2_ and IgE in asthmatic rats and suggest its preventive effect on asthma.

In a previous study, we demonstrated the effect of hydroethanolic extract of *C. longa* rhizome and curcumin on total and differential WBC counts, as well as serum oxidant/antioxidant biomarkers in an asthmatic rat model, which reflect the effect of the plant and its constituent curcumin, on systemic inflammation and oxidative stress [21]. However, in the current study, total and differential WBC count and BALF concentrations of PLA_2_, TP, cytokines (IFN-*γ* and IL-4), IgE, oxidant/antioxidant biomarkers (NO_2_, NO_3_, MDA, SOD, CAT, and thiol) in sensitized animals were assessed which reflected inflammatory, immunological, and oxidant changes in the lung of sensitized animals rather than systemic changes published in our previous paper. Sarkar et al. [7] also studied the effect of *C. longa* on total and differential WBC, nitrite, MDA, and myeloperoxidase (MPO) levels and percentage of intact and granulated mast cells in BALF, total protein in lung tissue homogenate, and serum IgG in sensitized animals. However, the inflammatory indices (PLA_2_ and TP) as well as the important immunological indices such as IgE, IL-4, IFN-*γ*, and IFN-*γ*/IL-4 ratio which reflect Th1 and Th2 activities were not measured in the mentioned study. In addition, in the present study, the effect of three concentrations of *C. longa* extract (0.75, 1.5, and 3 mg/ml equal to 0.15, 0.3, and 0.6 mg/kg/day) added to the drinking water of animals throughout 21 days of sensitization period was evaluated. However, in the study of Sarkar et al. [7], the effect of oral administration of 100 mg/kg of the extract, twice diurnal at 8.00 A.M. and 18.00 P.M., was examined which was much higher than the administered dose in our study.

The use of ELISA kits to detect inflammatory mediators and cytokines levels in the BALF appears to be a limitation of the current investigation. However, they do not involve any quality control samples, because the results of various groups were compared. The inclusion of a quality control sample would have no effect on the overall results. As seen in Figure 5, the variation in the obtained values was similarly quite minimal.

Concentration-dependent effects of the extract of *C. longa* on all investigated variables were observed in the present study. The effect of its two higher concentrations was higher than its low concentration. In addition, the effect of high concentration of *C. longa* was also higher than its medium concentration. The concentration-dependent effect of the extract of *C. longa* on total and differential WBC counts, inflammatory mediators, and immunological, antioxidant, and oxidant biomarkers in OVA-sensitized rats also supported antioxidant, anti-inflammatory, and immunomodulatory properties of this plant.

The results of the current study also indicated comparable or even higher effects of the extract of *C. longa* compared to those of dexamethasone. The effects of all concentrations of the extract of *C. longa* on IFN-*γ*, its two higher concentrations on total WBC, IL-4, and IFN-*γ*/IL-4 ratio, and its high concentration on eosinophils, lymphocytes, and monocytes percentages and BALF levels of TP, IgE, and thiol were higher than the effect of dexamethasone. Based on our results, a more specific immunomodulatory effect was observed for *C. longa* extract in comparison with dexamethasone. In asthmatic rats, treatment with *C. longa* extract lowered IL-4 levels while it increased IFN-levels and the IFN-/IL-4 ratio, showing decreased Th2 but higher Th1 activity and improved Th1/Th2 balance. Treatment with the extract of *C. longa* reduced IL-4 but increased IFN-*γ* level and IFN-*γ*/IL-4 ratio, indicating decreased Th2 but increased Th1 activity and enhanced Th1/Th2 balance in asthmatic rats. However, dexamethasone treatment reduced both IFN-*γ* and IL-4 and did not change IFN-*γ*/IL-4 ratio and Th1/Th2 balance. These results also indicated a possible preventive therapeutic effect for *C. longa* on asthma which was more potent and specific compared to that of dexamethasone.

## 5. Conclusion

The novel finding of the current investigation was that *C. longa* had a more specific immunomodulatory effect on Th1/Th2 balance in sensitized rats when compared to dexamethasone. In addition, the current study shows that *C. longa* has anti-inflammatory, immunological, and oxidant-antioxidant properties in an animal model of asthma. The findings of this study imply that *C. longa* therapy reduces airway inflammation by restoring the oxidant-antioxidant equilibrium. In addition, *C. longa* immunomodulatory properties may help to reduce lung inflammation.

As a result, our findings could point to complicated interactions between *C. longa* antioxidant, anti-inflammatory, and immunoregulatory effects in the treatment of asthma. The current data, along with those from prior studies, demonstrate that *C. longa* has anti-inflammatory, immunomodulatory, and antioxidant properties in sensitized rats, implying that it could be used to treat asthma. However, more research is needed to determine how *C. longa* affects asthmatic patients.

Finally, our findings showed that *C. longa* had a protective effect on lung inflammation, oxidative stress, and immunological indicators in sensitized animals. At the concentrations tested, *C. longa* had anti-inflammatory and antioxidant effects that were equivalent to or even better than dexamethasone, but its immunomodulatory action was more specific, resulting in an enhanced Th1/Th2 balance. These findings imply that *C. longa* has therapeutic promise in the treatment of asthma.

## Data Availability

The data used to support the findings of this study are included within the supplementary information file(s).
